# Salinomycin Exerts Anticancer Effects on PC-3 Cells and PC-3-Derived Cancer Stem Cells In Vitro and In Vivo

**DOI:** 10.1155/2017/4101653

**Published:** 2017-06-06

**Authors:** Yunsheng Zhang, Luogen Liu, Fang Li, Tao Wu, Hongtao Jiang, Xianxun Jiang, Xiaobo Du, Yi Wang

**Affiliations:** ^1^Clinical Research Institute, The Second Affiliated Hospital, University of South China, Hengyang 421001, China; ^2^College of Nursing, Hunan Polytechnic of Environment and Biology, Hengyang 421005, China; ^3^Department of Urology, The Second Affiliated Hospital, University of South China, Hengyang 421001, China; ^4^Department of Urology, The First People's Hospital of Yueyang, Yueyang 414000, China

## Abstract

Salinomycin is an antibiotic isolated from* Streptomyces albus *that selectively kills cancer stem cells (CSCs). However, the antitumor mechanism of salinomycin is unclear. This study investigated the chemotherapeutic efficacy of salinomycin in human prostate cancer PC-3 cells. We found that cytotoxicity of salinomycin to PC-3 cells was stronger than to nonmalignant prostate cell RWPE-1, and exposure to salinomycin induced G2/M phage arrest and apoptosis of PC-3 cells. A mechanistic study found salinomycin suppressed Wnt/*β*-catenin pathway to induce apoptosis of PC-3 cells. An in vivo experiment confirmed that salinomycin suppressed tumorigenesis in a NOD/SCID mice xenograft model generated from implanted PC-3 cells by inhibiting the Wnt/*β*-catenin pathway, since the total *β*-catenin protein level was reduced and the downstream target c-Myc level was significantly downregulated. We also showed that salinomycin, but not paclitaxel, triggered more apoptosis in aldehyde dehydrogenase- (ALDH-) positive PC-3 cells, which were considered as the prostate cancer stem cells, suggesting that salinomycin may be a promising chemotherapeutic to target CSCs. In conclusion, this study suggests that salinomycin reduces resistance and relapse of prostate tumor by killing cancer cells as well as CSCs.

## 1. Introduction

Prostate cancer is a commonly diagnosed cancer in male patients, which ranks the first in European and the last in Southern/Eastern Asian countries [1]. The primary treatment for patients is radiotherapy or prostatectomy. However, 30 to 50 percent of patients suffer from relapse, and the aberrant neoplastic growth resumes either locally or systemically [2]. Relapsed patients often require androgen deprivation therapy (ADT) [3]. However, ADT is accompanied with a high risk of death and dementia in prostate cancer patients [4].

New evidence suggests that CSCs play vital roles in the process of prostate cancer castration resistance and metastasis [5]. CSCs are capable of self-renewal and differentiation [6], which express specific surface antigens, such as ALDH [7]. Given the variety of therapies to which they are resistant, it is possible that CSCs exhibit generalized apoptosis resistance. Therefore, it might not be possible to identify therapies that target CSCs specifically [8]. However, this is not the case since it is possible to use unbiased screening strategies to systematically identify chemical compounds that target CSCs specifically [9].

In a screen of 16,000 chemicals to search for molecules that could selectively eradicate cancer stem cells (CSCs), Gupta et al. (2009) identified salinomycin as a novel anticancer agent with >100-fold higher potency than paclitaxel, a commonly used anti-breast cancer drug [10]. Salinomycin is a monocarboxylic polyether antibiotic isolated from* Streptomyces albus*. A recent study showed that salinomycin's action is comparable to that of nigericin (K^+^/H^+^ exchanger) while being distinct from the effects of valinomycin (K^+^ ionophore) [11]. In addition, several studies have shown that salinomycin has prooxidative [12], proautophagy [13], and proliferation-suppressive biological effects in human cancer cells [14]. However, it remains unclear whether these ionophoric properties and mechanisms could explain the observed specificity of salinomycin in CSCs and multidrug-resistant cancer cells.

This study was designed to investigate the chemotherapeutic efficacy of salinomycin in human prostate cancer PC-3 cells. Firstly, we examined the cytotoxicity of salinomycin in the nonmalignant prostate cell line RWPE-1 and prostate cancer cell line PC-3, which showed that PC-3 cell line was more sensitive to salinomycin than RWPE-1 cell line. Secondly, our studies showed that salinomycin inhibited proliferation and induced apoptosis of PC-3 cells. In order to understand the mechanism of action, we examined the Wnt/*β*-catenin pathway after salinomycin treatment in vitro and in vivo, which showed that the Wnt/*β*-catenin pathway was involved in the antitumor effect of salinomycin. Finally, we also showed that salinomycin triggered more apoptosis in ALDH-positive PC-3 cells than paclitaxel, suggesting that salinomycin may be a promising chemotherapeutic to target CSCs.

Based on the results, we postulated that salinomycin has a potential as a future chemotherapeutic, which may reduce resistance and relapse of prostate tumor by killing cancer cells as well as CSCs.

## 2. Materials and Methods

### 2.1. Cell Culture and Drugs

The nonmalignant prostate cell line RWPE-1 (ATCC, Manassas, VA, USA) and human prostate cancer cell line PC-3 (ATCC, Manassas, VA, USA) were cultured in recommended medium supplemented with 10% fetal calf serum (FBS; Gibco, Carlsbad, CA, USA), 100 units/mL penicillin, and 100 *μ*g/mL streptomycin in a humidified atmosphere with 5% CO_2_ at 37°C. Salinomycin (Sigma-Aldrich, St. Louis, MO, USA) was dissolved in dimethylsulfoxide (DMSO; Sigma-Aldrich, St. Louis, MO, USA). The 3-(4,5-dimethyl-2-thiazolyl)2,5-diphenyl-2H tetrazolium bromide (MTT; Sigma-Aldrich, St. Louis, MO, USA) assays were used to investigate cell viability. Briefly, RWPE-1 and PC-3 cells were seeded in 96-well plates and then treated with various concentrations of salinomycin. After incubation for 12 h, 24 h, or 48 h, cells were treated as previously described [15]. The absorbance was read at 570 nm using iMark Microplate Reader (Bio-Rad, Hercules, CA, USA).

### 2.2. Apoptosis Assessment

The 4,6-diamidino-2-phenylindole (DAPI) and acridine orange/ethidium bromide (AO/EB) staining were used to measure apoptosis. RWPE-1 and PC-3 cells were seeded in six-well plates and then treated with salinomycin (0 or 5.0 *μ*M) for 24 h. For DAPI staining, cells were washed three times with PBS (pH 7.4) and incubated with 0.5 *μ*g/mL DAPI staining for 5 min. For AO/EB staining, cells were incubated with AO and EB staining solution as previously described [16]. All of the images were viewed under a fluorescent microscope (Carl Zeiss, Jena, Germany).

### 2.3. Cell Cycle and Apoptosis Analyses

RWPE-1 and PC-3 cells were seeded in six-well plates and then treated with salinomycin (0, 2.0, or 5.0 *μ*M) for 24 h. For cell cycle analysis, cells were fixed and stained as previously described [17]. For apoptosis analysis, apoptosis and necrosis were evaluated by the annexin V-FITC/PI staining as previously described [17]. Samples were analyzed by a FACSCalibur (BD Biosciences, San Jose, CA, USA).

### 2.4. Determination of Mitochondrial Membrane Potential (Δ*ψ*m)

RWPE-1 and PC-3 cells were treated as described above. Δ*ψ*m was assessed using 5,5′,6,6′-tetrachloro-1,1′,3,3′-tetraethylbenzimidazole-carbocyanide iodine (JC-1; Beyotime, Shanghai, China) staining as described previously [18]. Red fluorescence represents JC-1 aggregates that appear in the mitochondria after potential-dependent aggregation. Green fluorescence represents JC-1 monomers that appear in the cytosol after depolarization of the mitochondrial membrane.

### 2.5. Western Blot

For western blot analysis, cells were harvested and extracted in lysis buffer (50 mM Tris (pH 7.4), 150 mM NaCl, 0.1% SDS, 1% NP-40, and 1 mM phenylmethylsulfonyl fluoride (PMSF)). The analysis was performed as described previously [17]. The primary antibodies used to detect apoptotic markers were Bcl-2 (Santa Cruz, California, CA, USA), Bax (Abcam, Cambridge, MA, USA), cytochrome C (Santa Cruz, California, CA, USA), PARP, cleaved PARP, caspase 3, cleaved caspase 3, caspase 7, cleaved caspase 7, caspase 9, and cleaved caspase 9 (all from CST, Danvers, MA, USA). The primary antibodies c-Myc, cyclin D1, and cyclin E1 (all from Abcam, Cambridge, MA, USA) were used to detect cell cycle proteins. *β*-Catenin, GSK3*β*, p-GSK3*β*, c-Myc, cyclin D1, and cyclin E1 (all from Abcam, Cambridge, MA, USA) were used to analyze the Wnt/*β*-catenin pathway. *β*-Actin (CST, Danvers, MA, USA) was used as an internal control.

### 2.6. Immunofluorescence

Immunofluorescence staining was performed to detect the translocation of *β*-catenin from the cytoplasm to nuclei in PC-3 cells. Briefly, the cells were cultured on coverslips and received salinomycin treatment. After 24 h, the cells were washed with PBS and processed in the same way as previously described [19]. The secondary antibodies conjugated with Alexa Fluor-647 (Abcam, Cambridge, MA, USA) were used in this experiment. The immunofluorescence-labeled cells were examined and analyzed by laser scanning confocal microscopy (Olympus, Tokyo, Japan).

### 2.7. Colony Formation Assay

RWPE-1 cells were seeded in six-well plates at a density of 500 cells per well, and PC-3 cells were seeded at a density of 50 cells per well. Cells were treated with salinomycin (0, 2.0, or 5.0 *μ*M) for 24 h. After incubation for an additional 2 weeks, the plates were fixed with chilled methanol and stained with Giemsa staining solution for 30 min. Visible colonies were manually calculated in triplicate.

### 2.8. In Vivo Treatment and Immunohistochemistry Assay

NOD/SCID IL2R*γ*c male mice were purchased from Beijing HFK BioScience Co., Ltd. Animal studies were approved by the animal ethics committee of South China University. PC-3 tumor xenograft mice model was established as described previously [20]. Mice were treated intraperitoneally (i.p.) daily for 3 weeks with either DMSO or salinomycin at a dose of 10 mg/kg/day/200 *μ*L. The tumor volume of mice was recorded every three days. The tumor volume was calculated according to the formula *A* × *B* × *B*/2, where *A* is the length of the tumor and *B* is the width. After 3 weeks, the mice were euthanized and xenografts were excised. Some fresh samples were analyzed with *β*-catenin, c-Myc, and cyclin D1 for western blot. The others were fixed in 10% formalin solution and paraffin-embedded and then stained with *β*-catenin, c-Myc, and cyclin D1 for immunohistochemistry.

### 2.9. Aldehyde Dehydrogenase Activity Assay

PC-3 cells were seeded in six-well plates and then treated with paclitaxel (100 nM) and salinomycin (5.0 *μ*M) for 24 h. For aldehyde dehydrogenase activity, ALDH-positive cells were identified using an ALDEFLUOR kit (Stemcell Technologies, Vancouver, Canada). Briefly, activated substrate was added to PC-3 cells that had been resuspended in ALDEFLUOR assay buffer. Half of the aforementioned mixtures were then immediately transferred to a tube containing diethylaminobenzaldehyde (DEAB) as a control. These mixtures were incubated for 30–60 min. ALDH^+^ cells were identified by using flow cytometry and analyzed by FlowJo 8.8.6 software (Tree Star Inc., Ashland, OR, USA).

### 2.10. Mammosphere Formation Assay

PC-3 cells were seeded in six-well ultralow attachment plates. The cells were allowed to grow and form spheres as previously described [21]. The primary PC-3 cells were incubated with paclitaxel (100 nM) and salinomycin (5.0 *μ*M) in mammosphere-forming conditions. After 5–7 days, the images of the mammosphere were viewed under a fluorescent microscope (Carl Zeiss, Jena, Germany).

### 2.11. Statistical Analysis

All statistical analyses were performed using SPSS 18.0 software (SPSS Inc., Chicago, IL, USA). A *P* value of less than 0.05 was considered statistically significant.

## 3. Results

### 3.1. Salinomycin Inhibits Cell Viability and Induces Apoptosis in PC-3 Cells

**Figure 1:**
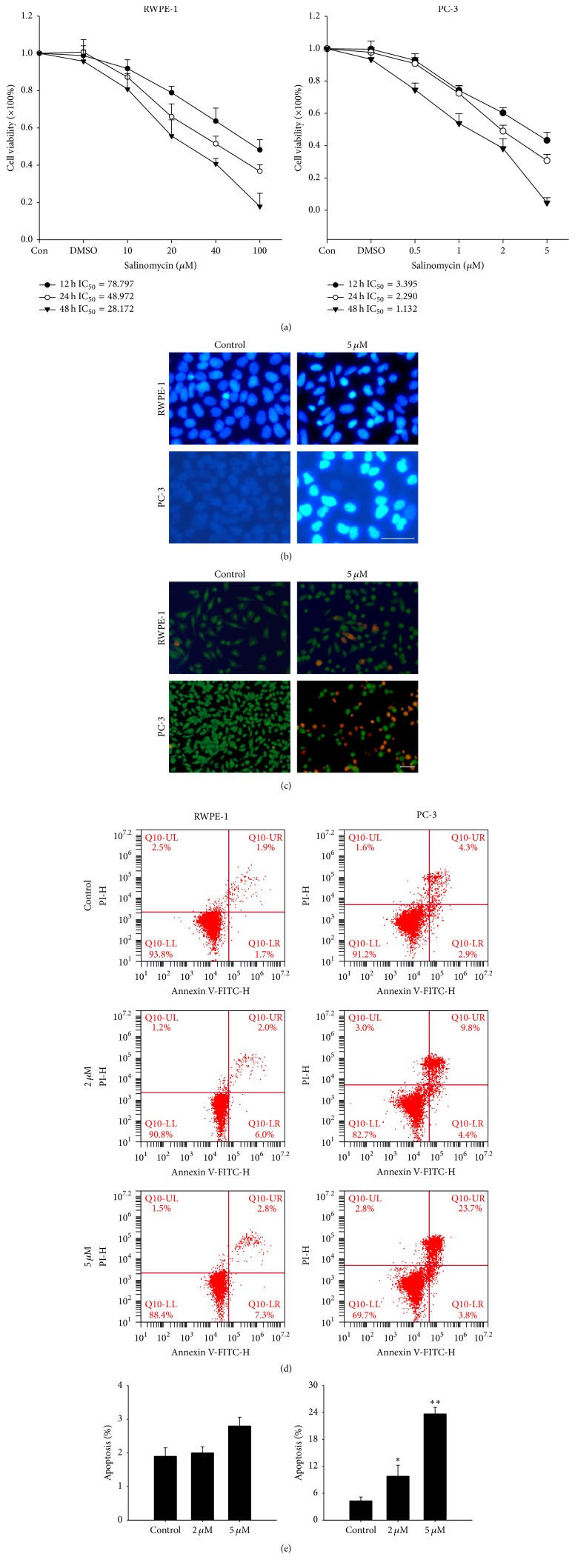
Salinomycin inhibits cell viability and induces apoptosis in PC-3 cells. (a) Cell viability was evaluated by MTT assays. The various concentrations of salinomycin were added to RWPE-1 and PC-3 cells for 12 h, 24 h, and 48 h. The IC_50_ values of salinomycin in PC-3 and RWPE-1 cells for the indicated time periods. (b) Chromatin condensation of RWPE-1 and PC-3 cells was induced by salinomycin. Cells were stained with DAPI and viewed under a fluorescence microscope. Scale bars: 10 *μ*m. (c) AO/EB staining of RWPE-1 and PC-3 cells, followed by viewing under a fluorescence microscope. Scale bars: 10 *μ*m. (d) Salinomycin induced RWPE-1 and PC-3 cells apoptosis. The annexin V-FITC/PI staining shows the rate of apoptosis after salinomycin treatment. (e) The histogram shows the apoptosis rate (%) of cells. Data are presented as means ± SDs (*n* = 3). ^*∗∗*^*P* < 0.01 and ^*∗*^*P* < 0.05 versus control.

In order to evaluate the chemotherapeutic efficacy of salinomycin, the cell viability was tested in prostate cancer cell line PC-3 and nonmalignant prostate cell line RWPE-1. After salinomycin treatment, the two cell lines showed a dose- and time-dependent decrease in cell viability. The IC_50_ values of PC-3 and RWPE-1 are indicated in Figure 1(a). At variable treatment times, the IC_50_ value of salinomycin in RWPE-1 cells was >20-fold higher than in PC-3 cells. These data demonstrate that salinomycin-mediated cytotoxicity is tumor-specific.

Furthermore, salinomycin induces apoptosis in PC-3 cells which was also stronger than in RWPE-1 cells. In our experiments, salinomycin triggered nucleus shrinkage in PC-3 cells was stronger (Figure 1(b)). Cell death was confirmed using AO/EB staining, which revealed more obvious apoptotic features in PC-3 cells (Figure 1(c)). Annexin V-FITC staining illustrated that salinomycin treatment resulted in apoptosis in PC-3 cells which was also stronger than in RWPE-1 cells (Figures 1(d) and 1(e)). These results indicate that salinomycin induces apoptosis in PC-3 cells and less cytotoxic effects on the normal cells.

### 3.2. Salinomycin Induces Mitochondria-Dependent Apoptosis in PC-3 Cells

**Figure 2:**
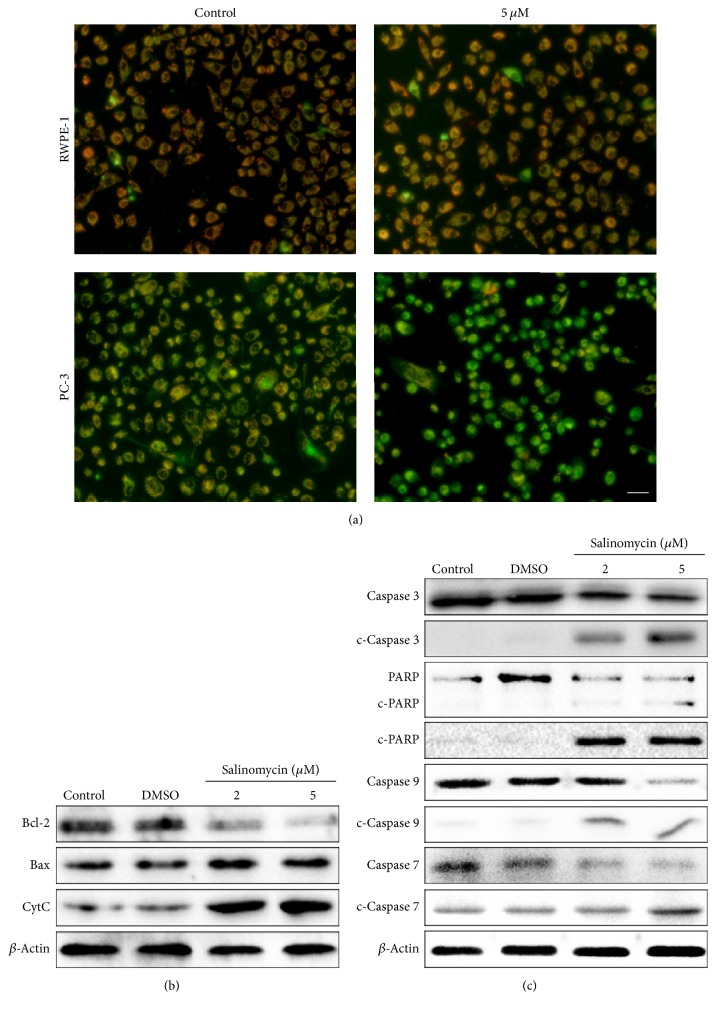
Salinomycin induces mitochondria-dependent apoptosis. (a) Salinomycin altered mitochondrial transmembrane potential (ΔΨm). Salinomycin was added to RWPE-1 and PC-3 cells for 24 h. Δ*ψ*m was assessed using JC-1 staining. Red fluorescence represents JC-1 aggregates. Green fluorescence represents JC-1 monomers. Scale bars: 10 *μ*m. There are more JC-1 aggregates in control cells, whereas JC-1 monomers formed after salinomycin treatment as a result of altered ΔΨm. (b) Salinomycin altered the expression of apoptosis-related proteins. Western blotting was used to see Bax, Bcl-2, and cytochrome C expression. (c) Salinomycin induced cleavage of caspases and PARP in PC-3 cells, indicative of apoptosis.

To characterize salinomycin-induced apoptosis, we measured mitochondrial membrane potential (ΔΨm) by JC-1 staining. JC-1 dye concentrates in the mitochondrial matrix and forms red fluorescent aggregates in normal cells. When ΔΨm is altered, JC-1 no longer accumulates and instead gets dispersed throughout the cells, forming green fluorescent monomers. As shown in Figure 2(a), RWPE-1 and PC-3 cells were treated with 5.0 *μ*M salinomycin for 24 h, which decreased the intensity of red fluorescence and increased green fluorescence staining in PC-3 cells. Meanwhile, PC-3 cells underwent morphologic changes. The cells shrank and turned round and became loosely arranged, and adhesion was attenuated gradually after incubation with salinomycin. These results indicate that salinomycin induces mitochondria-dependent apoptosis and ΔΨm loss in PC-3 cells and fewer effects on RWPE-1 cells.

Next, we detected the expression of proteins in the endogenous mitochondrial pathway after salinomycin treatment. The levels of Bcl-2 decreased in salinomycin-treated PC-3 cells, whereas the levels of Bax and cytochrome C increased (Figure 2(b)). Furthermore, salinomycin triggered the cleavage of caspases and PARP in PC-3 cells (Figure 2(c)). Our results indicate that salinomycin induces mitochondria-dependent apoptosis in PC-3 cells.

### 3.3. Salinomycin Inhibits Proliferation and Induces Cell Cycle Arrest in PC-3 Cells

**Figure 3:**
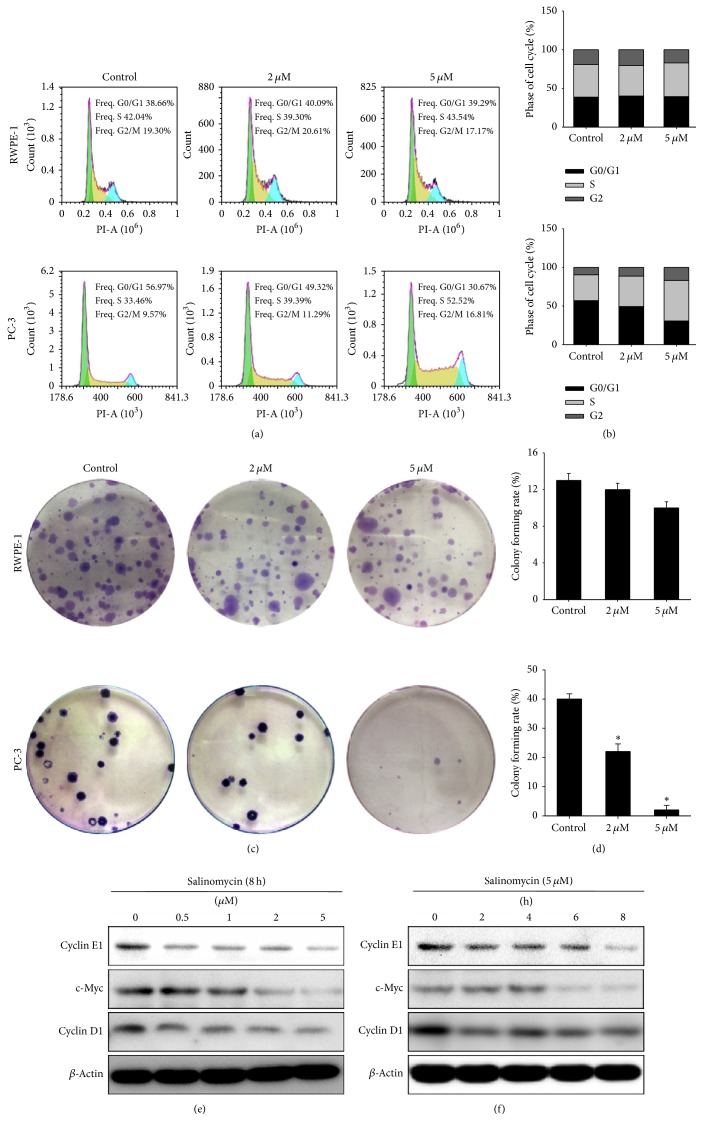
Salinomycin inhibits PC-3 cell proliferation and induces cell cycle arrest. (a) Salinomycin induced cell cycle arrest in PC-3 cells. The various concentrations of salinomycin were added to RWPE-1 and PC-3 cells for 24 h. Cell cycle progress was evaluated by flow cytometry. (b) The histogram shows the percentage of cells in G0/G1, S, and G2/M phases. (c) Salinomycin inhibits PC-3 cell proliferation. Salinomycin was added to RWPE-1 and PC-3 cells for 24 h. Cell proliferation was measured by colony formation assay. (d) The histogram shows the colony forming rate. Data are presented as means ± SDs (*n* = 3). ^*∗*^*P* < 0.01 versus control. (e and f) Changes in cell cycle regulatory proteins after salinomycin treatment for the indicated time periods and dose concentrations.

Given that salinomycin has anticancer effects, we further sought to determine whether salinomycin affected the cell cycle progress by flow cytometry. As shown in Figures 3(a) and 3(b), a substantial proportion of salinomycin-treated PC-3 cells were growth-arrested at the S checkpoint. The G2/M phase arrest was also observed in 5.0 *μ*M salinomycin-treated PC-3 cells. However, analysis of RWPE-1 cells had no obvious difference. The effect of salinomycin antiproliferation was also confirmed using colony formation assay (Figure 3(c)). PC-3 cell colony rates were 40%, 23%, and 2% at 0, 2, and 5 *μ*M, respectively. However, RWPE-1 cell colony rates were 13%, 12%, and 10% (Figure 3(d)). These data suggest that salinomycin inhibits proliferation by inducing S and G2/M phase cell cycle arrest in PC-3 cells and fewer effects on RWPE-1 cells.

We also evaluated the detailed mechanisms underlying salinomycin-induced cell cycle arrest; several proteins involved in S and G2/M arrest were measured by western blotting. Salinomycin treatment decreased protein levels of cyclin D1, cyclin E1, and c-Myc in a dose- and time-dependent manner, the crucial rate-limiting factors governing S and G2/M progress (Figures 3(e) and 3(f)). The loss of cyclin D1, cyclin E1, and c-Myc expression might be attributed to the cell cycle arrest.

### 3.4. Salinomycin Suppresses Wnt/*β*-Catenin Signaling Pathway

**Figure 4:**
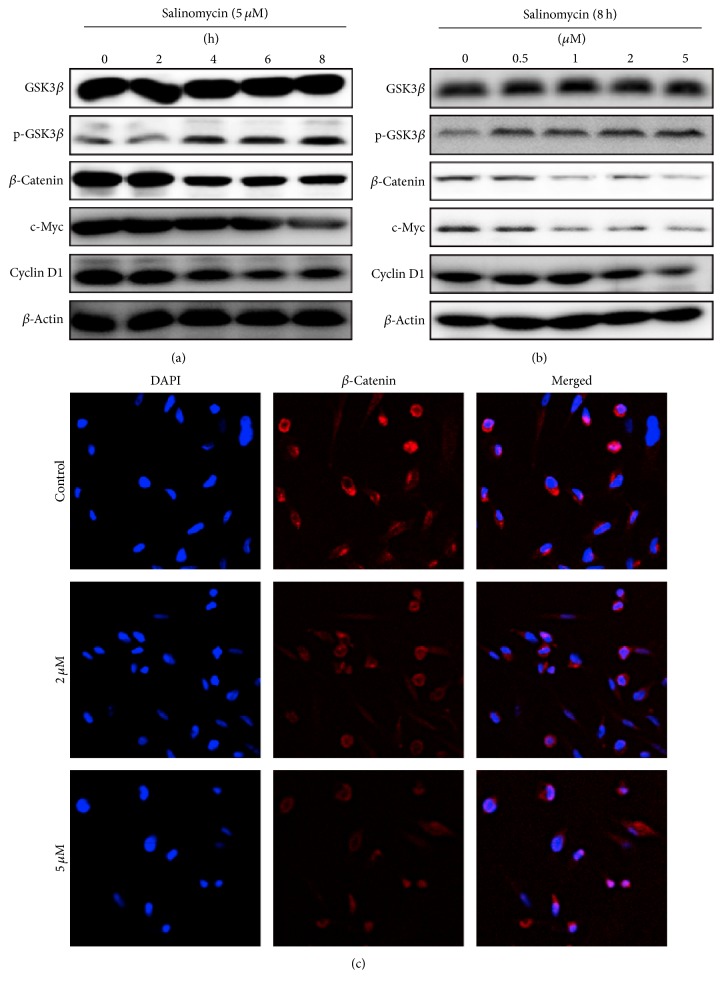
Salinomycin suppresses Wnt/*β*-catenin signaling pathway. (a and b) Salinomycin altered Wnt/*β*-catenin signaling pathway. PC-3 cells were treated with 5 *μ*M salinomycin for 0, 2, 4, 6, and 8 h or treated with 0, 0.5, 1, 2, and 5 *μ*M salinomycin for 8 h. The Wnt/*β*-catenin signaling pathway-related proteins were detected by western blot. (c) The immunofluorescence staining of *β*-catenin (red) in salinomycin-treated PC-3 cells; nuclei are stained with DAPI (blue). Scale bars: 10 *μ*m.

To determine the mechanism of salinomycin-mediated apoptosis, we analyzed the expression of proteins belonging to the Wnt/*β*-catenin pathway. Results showed that salinomycin treatment decreased the expression of *β*-catenin, c-Myc, and cyclin D1 in a dose- and time-dependent manner, while p-GSK3*β* was increased (Figures 4(a) and 4(b)). It is possible that reduced *β*-catenin, c-Myc, and cyclin D1 expression is caused by activation of GSK3*β* phosphorylation, as one of the possible mechanisms of salinomycin-mediated apoptosis.

To further characterize the effects of salinomycin on Wnt/*β*-catenin pathway, we tested the expression and translocation of *β*-catenin by immunofluorescence staining. As shown in Figure 4(c), strong positive staining was detectable in the control slides; however, weak staining was detectable after salinomycin treatment. Moreover, salinomycin seemingly enhanced *β*-catenin nuclear localization, which may be because apoptosis cells cause shriveling and staining was concentrated in the nucleus. These results suggest that salinomycin suppresses Wnt/*β*-catenin pathway in PC-3 cells.

### 3.5. Salinomycin Inhibits the Growth of PC-3 Cell Xenograft In Vivo

**Figure 5:**
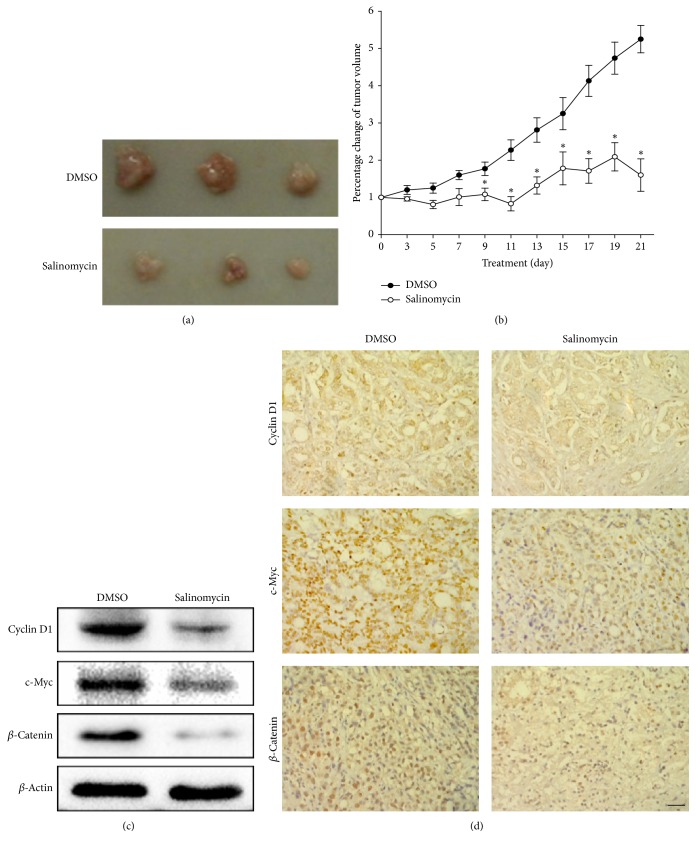
Salinomycin inhibits tumor growth in tumor xenografts. PC-3 tumor xenografts were treated intraperitoneally (i.p.) daily for 3 weeks with either DMSO or salinomycin. The tumor volume of mice was recorded every three days. (a) The excised tumors from DMSO or salinomycin group after 3 weeks. (b) The percentage change of tumor volume. The data are presented as means ± SEM. ^*∗*^*P* < 0.05 versus control. (c) The *β*-catenin, c-Myc, and cyclin D1 protein levels were examined by western blot in fresh xenograft tissues. (d) Representative immunohistochemical staining of *β*-catenin, c-Myc, and cyclin D1 in paraffin-embedded xenograft tissues. Scale bars: 10 *μ*m.

To evaluate the antitumor effects of salinomycin in vivo, we established the PC-3 tumor xenograft NOD/SCID IL2R*γ*c mice model. After 3 weeks, the excised xenografts were significantly decreased in salinomycin groups compared with DMSO groups (Figure 5(a)). Similarly, the observed percentage change of tumor volume in mice treated with salinomycin was significantly lower than that of DMSO groups. After 3 weeks, the DMSO groups had grown >525% of the original mean tumor volume (where the mean tumor volume at the start of treatment was 100%), whereas the mean tumor size in salinomycin groups was 160% (*P* < 0.01; Figure 5(b)).

As expected, salinomycin decreased *β*-catenin, c-Myc, and cyclin D1 expression, revealing a major mechanism of antitumor effect of salinomycin as demonstrated by western blot (Figure 5(c)) and immunohistochemistry (Figure 5(d)), respectively. Therefore, our data demonstrates that the Wnt/*β*-catenin pathway was involved in the antitumor effect of salinomycin.

### 3.6. Salinomycin Targets PC-3-Derived Cancer Stem Cells to Exert Antitumor Effects

**Figure 6:**
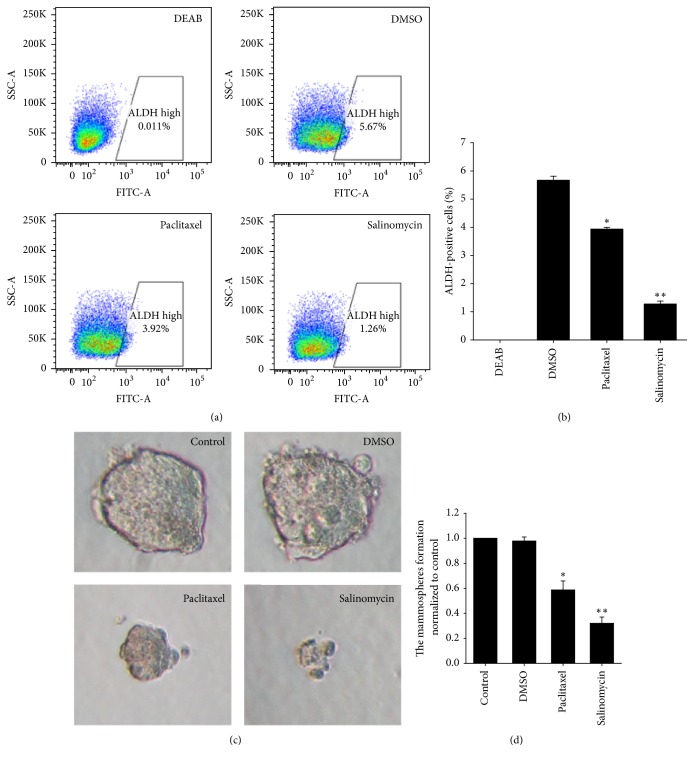
Salinomycin targets PC-3-derived CSCs to exert antitumor effects. (a) ALDH activity of PC-3 cells was analyzed by flow cytometry after salinomycin (5.0 *μ*M) or paclitaxel (100 nM) treatment. Diethylaminobenzaldehyde (DEAB), an ALDH inhibitor, was used as a negative control for ALDH staining. (b) The histogram shows the percentage of ALDH-positive cells. The data are presented as means ± SDs (*n* = 3). ^*∗∗*^*P* < 0.01 and ^*∗*^*P* < 0.05 versus DMSO. (c) The PC-3-derived CSCs mammospheres cultured in ultralow attachment plates and incubated with salinomycin (5.0 *μ*M) or paclitaxel (100 nM) for 7 days. Scale bars: 50 *μ*m. (d) The histogram shows the mammospheres formation normalized to control. The data are presented as means ± SDs (*n* = 3). ^*∗∗*^*P* < 0.01 and ^*∗*^*P* < 0.05 versus control.

Previous studies have shown that salinomycin can selectively kill cancer stem cells [10]. Therefore, we hypothesized that the target of the antitumor effect of salinomycin might be PC-3-derived cancer stem cells. Accordingly, we analyzed ALDH activity using an ALDEFLUOR kit. As shown in Figures 6(a) and 6(b), salinomycin (5.0 *μ*M) significantly diminished the ALDH-positive PC-3-derived CSC population by >78%. In contrast, paclitaxel (100 nM) diminished the ALDH-positive population of PC-3-derived CSCs by only 31%. This suggests that salinomycin might preferentially target PC-3-derived CSCs to exert antitumor effects.

In addition, CSCs can be enriched in mammospheres [20], which is based on the failure of cancer cells to survive in a serum-free medium [22]. So, we incubated PC-3 cells with salinomycin (5.0 *μ*M) or paclitaxel (100 nM) to evaluate the inhibitory effect of salinomycin on mammosphere formation. As shown in Figures 6(c) and 6(d), salinomycin suppressed the mammosphere formation of PC-3-derived CSCs. The results indicate that salinomycin targets PC-3-derived cancer stem cells to exert antitumor effects.

## 4. Discussion

Prostate cancer is a commonly diagnosed cancer in male patients. Despite the early diagnostic protocols and effective therapeutic interventions available, our understanding of prostate tumorigenesis remains limited, including knowledge of its origin and the factors and signaling pathways that regulate its initiation [23]. Prostate CSCs (PCSCs) are considered to be a reason for sustained prostate tumorigenesis [2]. Therefore, identifying specific drugs that target PCSCs might allow this cell type to be eliminated using targeted therapy, thereby improving therapeutic outcome [24].

Salinomycin was recently shown to have anticancer and anti-CSCs effects, as well as abilities to overcome multidrug resistance, based on xenografts in mice and pilot case reports of cancer patients [25]. However, the precise mechanism of salinomycin is unclear. Some studies have demonstrated that salinomycin inhibits CSC proliferation via the Wnt/*β*-catenin signaling pathway [26], induces apoptosis by causing the accumulation of reactive oxygen species [12, 25], and activates autophagy [27].

In this study, the cytotoxicity of salinomycin in human prostate cancer cell PC-3 and nonmalignant prostate cell RWPE-1 was evaluated, which showed that salinomycin-mediated cytotoxicity is tumor-specific. Cell morphology and nuclear area were altered significantly after salinomycin treatment, which led to apoptotic activation that is accompanied by the change of Bax/Bcl-2 ratio, the release of cytochrome C, the activation of PARP and caspases, and the loss of ΔΨm [28]. ΔΨm is a typical event in the endogenous mitochondrial pathway of apoptosis [29]. Bcl-2 family proteins and caspases are key regulators [30]. Therefore, we indicate that salinomycin induces mitochondria-dependent apoptosis in PC-3 cells.

Furthermore, salinomycin has been shown to interfere with the Wnt/*β*-catenin pathway in breast and prostate cancer cells [13]. The Wnt/*β*-catenin pathway is implicated in the maintenance of stem and progenitor cells in many cancer tissues, which has a role in prostate cancer according to a study of 14 prostate cancer samples [31]. Abnormal activation of this signaling pathway can initiate apoptosis independently and influence tumorigenesis [32]. Consistent with this, we examined the Wnt/*β*-catenin pathway after salinomycin treatment in PC-3 cells. Results showed that salinomycin decreased the expression of *β*-catenin, c-Myc, and cyclin D1 in a dose- and time-dependent manner, while p-GSK3*β* was increased. It is possible that the inhibition of Wnt/*β*-catenin signaling pathway is caused by activation of GSK3*β* phosphorylation after salinomycin treatment. Immunofluorescence staining also proved that salinomycin could suppress Wnt/*β*-catenin pathway in PC-3 cells. In vivo, salinomycin reduced xenografts tumor size and also suppressed Wnt/*β*-catenin pathway, which showed that the Wnt/*β*-catenin pathway was involved in the antitumor effect of salinomycin. Therefore, we revealed that Wnt/*β*-catenin pathway is one of the possible mechanisms of salinomycin-mediated apoptosis in PC-3 cells.

In addition, we also incubated PC-3 cells with salinomycin or paclitaxel to evaluate the inhibitory effect of salinomycin on mammosphere formation. As a common anticancer drug, paclitaxel can promote tubulin polymerization and stabilize microtubules from depolymerizing [33]. These results showed that salinomycin targeted PC-3-derived cancer stem cells to exert antitumor effects, suggesting that salinomycin may be a promising chemotherapeutic to target CSCs.

In conclusion, salinomycin inhibits proliferation of PC-3 cells, and the cytotoxicity is tumor-specific. Salinomycin induces mitochondria-dependent apoptosis in PC-3 cells. Furthermore, Wnt/*β*-catenin pathway is one of the possible targets by which salinomycin mediates apoptosis in PC-3 cells. In addition, salinomycin targets PC-3-derived cancer stem cells to prevent tumor relapse. Based on our results, we postulated that salinomycin has a potential as a future chemotherapeutic, which may reduce resistance and relapse of prostate tumor by killing cancer cells as well as CSCs.
